# Low heel ultrasound parameters predict mortality in men: results from the European Male Ageing Study (EMAS)

**DOI:** 10.1093/ageing/afv073

**Published:** 2015-07-09

**Authors:** Stephen R. Pye, Dirk Vanderschueren, Steven Boonen, Evelien Gielen, Judith E. Adams, Kate A. Ward, David M. Lee, György Bartfai, Felipe F. Casanueva, Joseph D. Finn, Gianni Forti, Aleksander Giwercman, Thang S. Han, Ilpo T. Huhtaniemi, Krzysztof Kula, Michael E. Lean, Neil Pendleton, Margus Punab, Frederick C. Wu, Terence W. O'Neill

**Affiliations:** 1 Arthritis Research UK Centre for Epidemiology, Institute of Inflammation and Repair, Faculty of Medical and Human Sciences, Manchester Academic Health Science Centre, The University of Manchester, Manchester, UK; 2 NIHR Manchester Musculoskeletal Biomedical Research Unit, Manchester, UK; 3 Department of Andrology and Endocrinology, Katholieke Universiteit Leuven, Leuven, Belgium; 4 Leuven University Division of Geriatric Medicine and Centre for Metabolic Bone Diseases, Katholieke Universiteit Leuven, Leuven, Belgium; 5 Radiology and Manchester Academic Health Science Centre, The Royal Infirmary, The University of Manchester, Manchester, UK; 6 MRC Human Nutrition Research, Elsie Widdowson Laboratory, Cambridge, UK; 7 Arthritis Research UK Epidemiology Unit, The University of Manchester, Manchester, UK; 8 Department of Obstetrics, Gynaecology and Andrology, Albert Szent-György Medical University, Szeged, Hungary; 9 Santiago de Compostela University, Complejo Hospitalario, Universitario de Santiago (CHUS), A Coruña, Spain; 10 Andrology Research Unit, Developmental and Regenerative Biomedicine Research Group, Manchester Academic Health Science Centre (MAHSC), The University of Manchester, Manchester, UK; 11 University of Florence, Florence, Italy; 12 University of Lund, Lund, Sweden; 13 Royal Free and University College Hospital Medical School, London, UK; 14 Imperial College, London, UK; 15 Medical University of Łódź, Łódź, Poland; 16 Human Nutrition, University of Glasgow, Glasgow, UK; 17 Geriatric Medicine, Salford Royal Hospital NHS Trust, Manchester, UK; 18 United Laboratories of Tartu University Clinics, Tartu, Estonia

**Keywords:** quantitative heel ultrasound, mortality, men, epidemiology, older people

## Abstract

**Background**: low bone mineral density measured by dual-energy x-ray absorptiometry is associated with increased mortality. The relationship between other skeletal phenotypes and mortality is unclear. The aim of this study was to determine the relationship between quantitative heel ultrasound parameters and mortality in a cohort of European men.

**Methods**: men aged 40–79 years were recruited for participation in a prospective study of male ageing: the European Male Ageing Study (EMAS). At baseline, subjects attended for quantitative ultrasound (QUS) of the heel (Hologic—SAHARA) and completed questionnaires on lifestyle factors and co-morbidities. Height and weight were measured. After a median of 4.3 years, subjects were invited to attend a follow-up assessment, and reasons for non-participation, including death, were recorded. The relationship between QUS parameters (broadband ultrasound attenuation [BUA] and speed of sound [SOS]) and mortality was assessed using Cox proportional hazards model.

**Results**: from a total of 3,244 men (mean age 59.8, standard deviation [SD] 10.8 years), 185 (5.7%) died during the follow-up period. After adjusting for age, centre, body mass index, physical activity, current smoking, number of co-morbidities and general health, each SD decrease in BUA was associated with a 20% higher risk of mortality (hazard ratio [HR] per SD = 1.2; 95% confidence interval [CI] = 1.0–1.4). Compared with those in higher quintiles (2nd–5th), those in the lowest quintile of BUA and SOS had a greater mortality risk (BUA: HR = 1.6; 95% CI = 1.1–2.3 and SOS: HR = 1.6; 95% CI = 1.2–2.2).

**Conclusion**: lower heel ultrasound parameters are associated with increased mortality in European men.

## Introduction

Data from observational studies suggest an association between low bone mineral density (BMD) assessed using bone densitometry and mortality including all-cause and cardiovascular disease [1–4]. The mechanism is uncertain though is thought in part to relate to adverse lifestyle factors linked with reduced BMD and also mortality. Quantitative ultrasound (QUS) is associated with an increased risk of fracture [5, 6]; however, less is known about other adverse outcomes including mortality. Lower heel ultrasound parameters have been associated with an increased mortality in some, though not all, observational studies in postmenopausal women [7–9]; however, to date, there are no data in men. We used data from a large population-based study of ageing in men to determine the association between QUS parameters and mortality (both all-cause and attributable to cardiovascular disease [CVD]), and whether the association can be explained by lifestyle or adverse health factors.

## Methods

### Subjects and study design

The subjects included in this analysis were recruited for participation in the European Male Ageing Study (EMAS). Details concerning the study design and recruitment have been previously described [10]. Briefly, an age-stratified probability sample of 3,369 men aged 40–79 (mean ± SD: 60 ± 11) years were recruited from population registers in eight European centres (Florence, Italy; Leuven, Belgium; Malmö, Sweden; Manchester, UK; Santiago de Compostela, Spain; Lódz, Poland; Szeged, Hungary; Tartu, Estonia). The choice of sampling frame was based on availability and representativeness of the local adult population within each centre and included primary care registers (UK), population registers and also electoral registers. The participation rate overall was 45% though there was some variation in rate by centre [10]. Subjects completed a postal questionnaire and then attended a research clinic for further assessments which included a single fasting morning (before 1,000 h) venous blood sample [10]. Subjects were subsequently invited to attend a follow-up assessment and completed another postal questionnaire (a median of 4.3 years later (range 3.0–5.7 years)). Those who did not reply after the initial contact were sent a further reminder. Ethical approval for the study was obtained in accordance with local institutional requirements in each centre. All subjects provided written informed consent.

### Assessments

The postal questionnaire included questions concerning current smoking, frequency of alcohol consumption, general health and whether subjects were currently being treated for a range of 16 medical conditions (heart condition, hypertension, bronchitis, asthma, peptic ulcer, epilepsy, diabetes, liver condition, diseases of the pituitary, testis, prostate, adrenal glands and thyroid, cancer, stroke and fracture since the age of 25). Physical activity was assessed using the Physical Activity Scale for the Elderly (PASE) [11]. Serum 25-hydroxyvitamin D (25(OH)D) levels were determined using a radioimmunoassay (RIA kit; DiaSorin, Stillwater, MN, USA) as described previously [12]. Information was also collected on current medications. Height and body weight were measured in standardised methods and body mass index (BMI) was calculated as weight (kg) divided by the square of height (m).

### QUS of the heel

QUS of the left heel was performed with the Sahara Clinical Sonometer (Hologic, Inc., Bedford, MA, USA) using a standardised protocol in all centres. Outputs included broadband ultrasound attenuation (BUA measured in dB/MHz) and speed of sound (SOS measured in m/s). Short-term precision was measured by performing duplicate measurements in 20 randomly selected subjects from one centre (Leuven, Belgium). The *in vivo* CVs were 2.8 and 0.3% for BUA and SOS, respectively. Repeat measurements (*n* = 10) were performed on a roving phantom at each of the eight centres. Standardised CVs (SCVs) [13] for within machine variability ranged by centre: for SOS, from 1.0 to 5.6%, and BUA from 0.7 to 2.7%. SCVs for between machine variability were 4.8% for BUA and 9.7% for SOS.

### Mortality

Deaths that occurred during the follow-up period were initially ascertained either through direct contact by relatives on receipt of the postal questionnaire or, if this was not returned, by further enquiry made to ascertain the subject's vital status. The enquiry procedure varied between centres and included review of medical records/death registers and telephone follow-up. Deaths were verified from: death certificates (28%), death registers (37%) and medical/hospital records (24%). Eleven per cent of deaths were unverified and information from the family member/contact person was the only source. Deaths were categorised where possible as being due to CVD, cancer or other causes, using where possible the same source as that used to verify the death. Subjects who did not reply to the follow-up postal questionnaire and for whom no further information was available were classified as ‘lost to follow-up’.

### Statistical analysis

Subjects contributed follow-up time (person-years) from the date of participation in the baseline survey to the point of last contact, which was either the date of the follow-up assessment or the date of death. All deaths occurring up to 31 December 2008 were included in this analysis. Body mass index (BMI) was treated as a continuous variable and also categorised into three groups: <20, 20–30 and 30 kg/m^2^ and over. Smoking status was categorised as current, previous and never. Poor general health was categorised as fair/poor versus excellent/very good/good. Co-morbidity was categorised as either none or one or more reported morbid conditions. The QUS parameters were *z*-scored (per standard deviation [SD]), categorised also into quintiles and further dichotomised into lowest and higher quintiles 2–5.

Differences in baseline characteristics between those who died and survivors were assessed using the *t*-test and the Wilcoxon rank-sum test as appropriate based on the underlying distribution of the variables. Cox proportional hazard models were used to assess the association between putative confounders and mortality (all-cause and CVD) adjusting for age and centre. Hazard models were also used to assess the association between the QUS parameters (per SD, categorised into quintiles and dichotomised as lowest quintile versus higher quintile) and mortality (all-cause and CVD) initially unadjusted, then serially adjusting for age and centre, then factors found to be associated with mortality in the earlier analysis. Sub-analyses were conducted comparing subjects under the age of 60 to those aged 60 and over, and also excluding deaths in the first 6 months and also the first year of follow-up. The results are reported as hazard ratios (HR) and 95% confidence intervals (CIs). For all models, the assumption of proportional hazards was tested using the Schoenfeld residuals. All statistical tests were performed using STATA version 11.2 (http://www.stata.com).

## Results

### Subjects

A total of 3,244 men had complete baseline QUS data. During a median of 4.3 years of follow-up (range 3.0–5.7), comprising 12,072 person-years, there were 185 deaths (5.7%). No information about vital status was obtained in 411 subjects. The baseline characteristics of the cohort, stratified by vital status at the end of the follow-up period, are presented in Table 1. Those who died were older (69.3 versus 59.2 years), had a lower weight (79.6 versus 83.6 kg) and also had a lower PASE score. A greater proportion of those who died smoked, consumed alcohol less frequently, had at least one co-morbid condition and were in fair or poor general health. Mean BUA (74.4 versus 80.4 dB/MHz) and SOS levels (1,538.8 versus 1,551.1 m/s) were lower in those who died compared with survivors.

**Table 1.** Baseline characteristics by vital status

	Survived (*n* = 2,648)	Died (*n* = 185)	*P*_diff_ value
	Mean (SD)		
Age (years)	59.2 (10.6)	69.3 (8.3)	<0.001
Height (cm)	174.0 (7.2)	170.1 (6.9)	<0.001
Weight (kg)	83.6 (13.4)	79.6 (15.9)	<0.001
BMI (kg/m^2^)	27.6 (3.9)	27.5 (4.8)	NS
PASE score	200.6 (88.8)	141.5 (90.3)	<0.001
QUS BUA (dB/MHz)	80.4 (17.8)	74.4 (20.3)	<0.001
QUS SOS (m/s)	1,551.1 (31.7)	1,538.8 (34.1)	<0.001
	%	%	
Current smoker (yes versus no)	20.0	26.5	0.03
Alcohol consumption, >1 day/week	57.6	41.4	<0.001
≥1 co-morbidities versus none	49.7	84.9	<0.001
Fair/poor general health	29.5	63.0	<0.001

*P*_diff_ using *t*-test or Wilcoxon rank-sum test.

### Mortality

Of the 185 deaths, 70 (37.8%) were considered related to CVD and 71 (38.3%) related to cancer. Seven (3.8%) deaths were attributable to both CVD and cancer. Of the remaining deaths, 33 (17.8%) were from other causes and in 18 (9.7%) the cause could not be determined. After adjustment for centre, increasing age (per year) was associated with a 10% higher risk in all-cause mortality (Table 2). After adjustment for age and centre, increasing physical activity, as measured by PASE score, was associated with a lower all-cause mortality. In contrast, current smoking, the presence of at least one co-morbid condition and fair/poor general health were associated with higher all-cause mortality. There was no relationship between BMI or alcohol consumption and mortality after adjustment for age and centre. Similar findings were observed for deaths that were related to CVD disease (Table 2).

**Table 2.** Influence of age, anthropometry, lifestyle and general health on all-cause and cardiovascular-related mortality

	All-cause mortality	CVD-related mortality
	Hazard ratio^a^ (95% CI)	
Age (years)^b^	1.1 (1.09–1.12)***	1.1 (1.09–1.16)***
BMI (kg/m^2^)	0.97 (0.9–1.0)	1.0 (0.98–1.1)
BMI (kg/m^2^)		
<20	2.4 (0.99–6.0)	1.7 (0.2–12.7)
20–30	Referent	Referent
30+	0.9 (0.6–1.3)	1.3 (0.8–2.2)
PASE score (per 100)	0.7 (0.5–0.8)***	0.5 (0.4–0.8)**
Current smoker (yes versus no)	2.3 (1.7–3.3)***	3.0 (1.7–5.2)***
Alcohol consumption/week		
None	1.4 (0.9–2.2)	1.2 (0.6–2.6)
<1 day	0.9 (0.6–1.4)	0.9 (0.4–1.8)
1–2 days	Referent	Referent
3–4 days	0.7 (0.4–1.4)	0.5 (0.1–1.9)
5–6 days	0.6 (0.3–1.5)	0.3 (0.04–2.2)
Every day	1.2 (0.7–2.0)	1.1 (0.5–2.8)
Co-morbidities		
None	Referent	Referent
One or more	2.3 (1.5–3.5)***	4.5 (1.8–11.5)**
General health		
Excellent/very good/good	Referent	Referent
Fair/poor	2.3 (1.6–3.2)***	3.9 (2.1–7.5)***

^a^Adjusted for age and centre except ^b^adjusted for centre.

** *P* < 0.01.

*** *P* < 0.001.

### QUS and mortality

Table 3 shows the association between QUS parameters and all-cause mortality. BUA (per SD decrease) was associated with a higher risk of mortality (HR = 1.4 per unit change in BUA), an effect that remained significant after adjustment for age, centre, smoking, physical activity, co-morbidities and general health (HR = 1.2; 95% CI = 1.0–1.4). A similar association was observed with SOS, though after adjustment for lifestyle and general health factors the CI just failed to exclude unity (HR = 1.2; 95% CI = 0.99–1.4). When the QUS parameters were categorised into quintiles, compared with those in the highest quintile of BUA or SOS, the risk of death was highest in those in the lowest quintiles. This was significant after adjustment for age and centre though the effect was attenuated and became non-significant after further adjustment for lifestyle and general health factors. There was no clear dose–response effect. After further categorisation, compared with those in quintiles 2–5, men in the lowest quintile of both BUA and SOS were at significantly higher risk of mortality—an effect that persisted after adjustment for confounders (BUA, HR = 1.6; 95% CI = 1.1–2.3; SOS, HR = 1.6; 95% CI = 1.2–2.2). This effect persisted after further adjustment for serum 25(OH)D (data not shown). Broadly similar results were evident for those deaths considered related to CVD (see the Supplementary data, Table Appendix S1, available in Age and Ageing online). Excluding men who died in the first 6 months (*n* = 10) or first year of follow-up (*n* = 23) made no difference to the results (data not shown).

**Table 3.** Influence of QUS parameters on all-cause mortality

	Unadjusted	Adjusted for age and centre	Adjusted for age, centre, current smoking, physical activity, co-morbidities and general health
	Hazard ratio (95% CI)		
BUA (per SD decrease)	1.4 (1.2–1.6)***	1.3 (1.1–1.5)**	1.2 (1.0–1.4)*
BUA quintiles: (dB/MHz)			
5: >95.0	Referent	Referent	Referent
4: 83.9–95.0	1.2 (0.7–1.9)	1.0 (0.6–1.7)	0.9 (0.5–1.6)
3: 74.8–83.8	1.3 (0.8–2.2)	1.1 (0.7–1.9)	1.1 (0.6–1.9)
2: 65.5–74.7	1.0 (0.6–1.8)	1.0 (0.6–1.7)	0.8 (0.5–1.5)
1: <65.5	2.3 (1.5–3.6)***	1.8 (1.2–2.9)*	1.5 (0.9–2.5)
BUA quintiles: (dB/MHz)			
5/4/3/2: ≥65.5	Referent	Referent	Referent
1: <65.5	2.0 (1.5–2.7)***	1.8 (1.3–2.5)***	1.6 (1.1–2.3)**
SOS (per SD decrease)	1.5 (1.3–1.8)***	1.3 (1.1–1.5)**	1.2 (0.99–1.4)
SOS quintiles: (m/s)			
5: >1,574.7	Referent	Referent	Referent
4: 1,555.6–1,574.7	1.6 (0.9–2.7)	1.4 (0.8–2.4)	1.4 (0.8–2.5)
3: 1,538.7–1,555.5	1.3 (0.7–2.2)	1.1 (0.6–1.8)	0.9 (0.5–1.6)
2: 1,523.6–1,538.6	1.3 (0.8–2.3)	1.0 (0.6–1.7)	0.8 (0.5–1.5)
1: <1,523.6	3.1 (1.9–5.0)***	2.0 (1.2–3.3)**	1.6 (0.9–2.8)
SOS quintiles: (m/s)			
5/4/3/2: ≥1,523.6	Referent	Referent	Referent
1: <1,523.6	2.4 (1.8–3.3)***	1.8 (1.3–2.5)***	1.6 (1.2–2.2)**

* *P* < 0.05.

** *P* < 0.01.

*** *P* < 0.001.

## Discussion

In this population-based sample of middle aged and older European men, lower BUA and SOS were associated with a higher risk of mortality. Those in the lowest quintile of both BUA and also SOS measurements had a 60% increased risk of all-cause mortality—an effect that persisted after adjustment for age, centre, lifestyle and general health factors. We observed similar results for CVD-related mortality. In keeping with many other studies, we identified smoking, low physical activity, number of co-morbidities and poor general health as predictors of mortality.

The relationship between low BMD and mortality has been confirmed in many prospective studies. Using data from the Study of Osteoporotic Fractures (SOF), Browner *et al.* [1] reported an age-adjusted relative risk of 1.19 per each SD reduction in proximal radius BMD as measured by single-photon absorptiometry (95% CI = 1.04–1.36). The authors suggested that low BMD was probably a marker of the cumulative effect of many other variables that influence mortality. Indeed, the association they observed was no longer statistically significant in multivariable analyses that included general health measures (such as self-reported health status, muscle strength and current physical activity). Interestingly, though in a model that included other predictors of mortality that are less linked to bone status (diabetes mellitus, hypertension and previous stroke), the association remained significant [1]. In a further study, Johansson *et al.* [2] also reported a 1.19-fold increase in mortality (95% CI = 1.02–1.39) per 1 SD reduction in BMD as measured by dual-photon absorptiometry at the calcaneus in women after adjustment for blood pressure and serum lipids. The authors considered that BMD is a predictor of survival independent of other diseases, and a better predictor of death than blood pressure or cholesterol. Kado *et al.* [3] studied the relationship between mortality and the rate of bone loss as measured by DXA, and found that women losing >2% per year at the hip had a mortality risk about three times greater than those losing <1%. Trivedi *et al.* [4] observed similar findings in men. After adjusting for major cardiovascular risk factors, they observed a 30% reduction in mortality per 1 SD increase in DXA assessed BMD at the hip. In a further Danish cohort [14], using single-photon absorptiometry at the distal forearm, for each decrease of 1 SD in BMD, the risk of all-cause mortality increased by 41% (relative risk [RR] = 1.4; 95% CI = 1.0–1.9).

Data from many studies suggest that BMD is not only related to all-cause mortality but also to cardiovascular mortality [1, 3, 4, 8, 15–18], vascular morbidity [19–21], cancer [17] and deaths from other causes [17]. The reasons for this are unclear, but the non-specificity in relation to mortality suggests that low BMD may be a marker of poor overall general health.

QUS parameters have been shown to predict fracture [5, 6, 22–25]; however, in contrast to findings in relation to BMD, there are relatively fewer data linking QUS parameters to mortality and no data in men. Bauer *et al.* [7] looked at the association between BUA measured at the calcaneus and total mortality in the Study of Osteoporotic Fractures. There was a positive association with an HR of 1.16 (95% CI = 1.07–1.26) for all-cause mortality. Pinheiro *et al.* [8] have followed a small cohort of 275 elderly women for 5 years. Each 1 SD reduction in stiffness index at baseline was significantly associated with total mortality (HR = 1.57; 95% CI = 1.10–2.47). Our data are broadly consistent with these findings and extend them to a male cohort and suggest that low QUS parameters in men are linked with an excess mortality. There was an increased risk of both all cause and also mortality from CVD, in keeping with data from the European Prospective Investigation into Cancer and Nutrition (EPIC)-Norfolk which reported that low heel ultrasound parameters predicted incident heart failure in men [26]. The association with mortality persisted after adjustment for lifestyle and also adverse health factors including co-morbidity suggesting that these factors do not explain the observed excess risk, though some caution is required in interpreting these data—it is not possible for example to exclude residual confounding or exposure misclassification as a possible explanation.

The main strengths of our study were that it was prospective, population-based and included data on a range of putative confounders including lifestyle factors and co-morbidities. There are a number of methodological limitations to be considered. In our study, the overall loss to follow-up rate was 9.9%. Compared with participants, those lost to follow-up had lower 25(OH)D levels, physical function and mental processing speed [27]. Our findings may therefore underestimate the true mortality experience of the full cohort. However, health status did not differ in a systematic fashion between subjects who participated and those lost to follow-up in relation to the QUS measurements. Thus, losses to follow-up are unlikely to have influenced the main findings in relation to the relationship between QUS parameters and mortality. The loss to follow-up rate varied across the participating centres (4.4–16.3%), which may reflect socio-cultural differences in the populations studied and also the extent to which the losses to follow-up were traced at different centres. Adjusting for the effect of centre in the analysis did not, however, alter the main findings. Furthermore, when the analysis was restricted to centres with >85% follow-up, the results remained unchanged.

In summary, in this population-based sample of middle aged and older European men, lower quantitative ultrasound parameters of the heel are associated with an increased risk of mortality. This was not explained by the occurrence of lifestyle factors or the presence of co-morbidities.

**Key points**

- Those in the lowest quintile of QUS measurements had a 60% increased risk of all-cause mortality.
- The increase in risk of mortality persisted after adjustment for lifestyle factors and the presence of co-morbidities.
- Smoking, low physical activity, number of co-morbidities and poor general health were also found to be predictors of mortality.

## Conflicts of interest

None declared.

## Funding

This work was supported by the Commission of the European Communities Fifth Framework Programme “Quality of Life and Management of Living Resources” (grant number QLK6-CT-2001-00258) and by Arthritis Research UK (grant number 20380). This report includes independent research supported by the National Institute for Health Research Biomedical Research Unit Funding Scheme. The views expressed in this publication are those of the author(s) and not necessarily those of the NHS, the National Institute for Health Research or the Department of Health. Dr K.A.W. is a senior research scientist working within the Nutrition and Bone Health Core Program at MRC Human Nutrition Research, funded by the UK Medical Research Council (grant number U105960371). Dr D.V. is a senior clinical investigator supported by the Clinical Research Fund of the University Hospitals Leuven, Belgium. The financial sponsors played no role in the design, execution, analysis and interpretation of data, or writing of this study.

## Supplementary data

Supplementary data mentioned in the text are available to subscribers in *Age and Ageing* online.

Supplementary Data
