# Supplementary material for: Low heel ultrasound parameters predict mortality in men: results from the European Male Ageing Study (EMAS)
Source: Age Ageing. 2015 Jul 9;44(5):801–7. doi: 10.1093/ageing/afv073 (PMC4547925; doi:10.1093/ageing/afv073)
Supplement: Supplementary Data [file supp_44_5_801__index.html]

Low heel ultrasound parameters predict mortality in men: results from the European Male Ageing Study (EMAS) — Supplementary Data 

# Low heel ultrasound parameters predict mortality in men: results from the European Male Ageing Study (EMAS)

## Supplementary Data

Supplementary Data

- Supplementary Data - Docx file
