# Supplementary material for: Low heel ultrasound parameters predict mortality in men: results from the European Male Ageing Study (EMAS)
Source: Age Ageing. 2015 Jul 9;44(5):801–7. doi: 10.1093/ageing/afv073 (PMC4547925; doi:10.1093/ageing/afv073)
Supplement: Supplementary Data [file supp_afv073_afv073supp.docx]

**Supplementary Material**

Appendix 1. Influence of QUS parameters on CVD-related mortality.

|  | Unadjusted | Adjusted for age and centre | Adjusted for age, centre, current smoking, physical activity, co-morbidities and general health |
| --- | --- | --- | --- |
|  | Hazard Ratio (95%CI) | | |
| BUA (per SD decrease) | **1.7 (1.3, 2.2)***** | **1.5 (1.2, 2.0)**** | **1.4 (1.1, 1.8)*** |
| BUA quintiles: (dB/MHz)  5: > 95.0  4: 83.9 – 95.0  3: 74.8 – 83.8  2: 65.5 – 74.7  1: < 65.5 | Referent  0.5 (0.2, 1.5)  1.1 (0.5, 2.5)  1.4 (0.6, 3.0)  **2.5 (1.2, 5.0)*** | Referent  0.5 (0.2, 1.3)  0.9 (0.4, 2.0)  1.2 (0.5, 2.7)  1.9 (0.9, 4.0) | Referent  0.5 (0.2, 1.5)  0.9 (0.4, 2.4)  1.4 (0.6, 3.5)  1.7 (0.7, 3.9) |
| BUA quintiles: (dB/MHz)  5/4/3/2: ≥ 65.5  1: < 65.5 | Referent  **2.4 (1.5, 4.0)***** | Referent  **2.1 (1.3, 3.5)**** | Referent  **1.8 (1.0, 3.1)*** |
| SOS (per SD decrease) | **2.0 (1.5, 2.7)***** | **1.6 (1.2, 2.0)**** | **1.4 (1.1, 1.9)*** |
| SOS quintiles: (m/s)  5: > 1574.7  4: 1555.6 – 1574.7  3: 1538.7 – 1555.5  2: 1523.6 – 1538.6  1: < 1523.6 | Referent  2.4 (0.9, 6.3)  1.2 (0.4, 3.5)  2.0 (0.8, 5.4)  **5.7 (2.4, 13.6)***** | Referent  2.2 (0.8, 5.9)  0.9 (0.3, 2.8)  1.4 (0.5, 3.9)  **3.2 (1.3, 7.9)*** | Referent  2.7 (0.9, 8.6)  1.0 (0.3, 3.5)  1.5 (0.4, 4.8)  **3.0 (1.0, 8.8)*** |
| SOS quintiles: (dB/MHz)  5/4/3/2: ≥ 1523.6  1: < 1523.6 | Referent  **3.4 (2.1, 5.5)***** | Referent  **2.3 (1.4, 3.8)***** | Referent  **2.0 (1.2, 3.3)*** |

***p<0.05 **p<0.01 ***p<0.001**
